# Post-operative Hypertension following Correction of Flexion Deformity of the Knees in a Spastic Diplegic Child: A Case Report

**DOI:** 10.5704/MOJ.1611.006

**Published:** 2016-11

**Authors:** Mohan Vipin, DM Sujendra, VK Imthiaz, Hitesh-Shah

**Affiliations:** Department of Orthopaedic Surgery, Manipal University Kasturba Medical College, Manipal, India

**Keywords:** Hypertension, Post operation, Cerebral palsy, flexion deformity of the knee, Femoral shortening

## Abstract

An adolescent boy with spastic diplegic cerebral palsy presented with crouch gait. He had bilateral severe flexion deformities of knees and hips. He was treated with single event multilevel surgery for the correction of deformities. Surgical procedures included bilateral adductor release, iliopsoas lengthening, bilateral femoral shortening and patella plication. Persistent hypertension was noted in the post-operative period. All causes of secondary hypertension were ruled out. Having persistent hypertension following the femoral shortening procedure is unusual. Antihypertensive medication controlled his blood pressure 15 months after surgery. Hypertension following correction of knee flexion deformity and limb lengthening is well known. Hypertension has not been described with the shortening osteotomy of the femur. Hypertension is a rare complication following the corrective surgery for the treatment of crouch gait. Blood pressure should be monitored during the post-operative period to detect such a rare complication.

## Introduction

Crouch gait is the most energy expensive gait pattern in cerebral palsy^1^. It is characterized by persistent flexion of the knee throughout the stance phase. The treatment with femoral shortening is reported to be quite effective^1^. Neurovascular complications are less common with femoral shortening osteotomy compared to distal femoral extension osteotomy^1^. Hypertension following knee flexion deformity correction has been described in literature^2, 3^. To the best of our knowledge hypertension has not been described with the shortening osteotomy of the femur. We report an occurrence of hypertension following the deformity correction with femoral shortening osteotomy.

## Case Report

A 15 years old South Indian boy with spastic cerebral palsy presented with walking crouch gait. He was born at full term normal delivery. The parents were not consanguineous. Both parents were normal. Mother did not take any medication before and during pregnancy for medical illness. No perinatal insult was noted. The developmental milestones were delayed. The child started standing around 1.5 years of age and walking around 2.5 years of age. No history of regression of developmental milestones was noted. The child was not on any treatment before presenting to us. He did not have any medical illnesses and was not on any medication before the presentation. On examination, he had spasticity of all four limbs (lower limbs were involved more than the upper limbs). The diagnosis of cerebral palsy was made on clinical grounds.

**Fig. 1:**
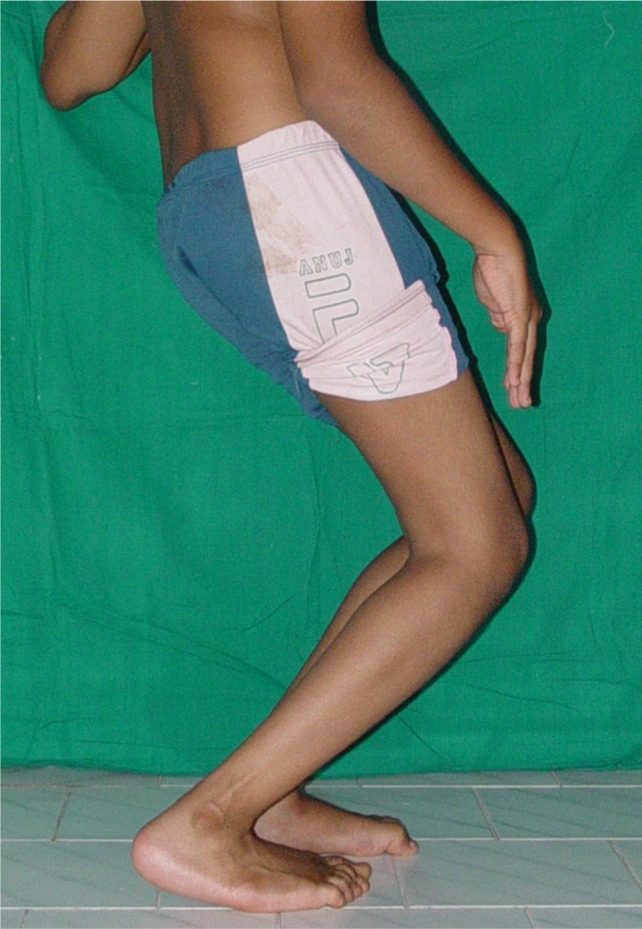
Pre-op standing photograph showing severe crouch gait.

**Fig. 2:**
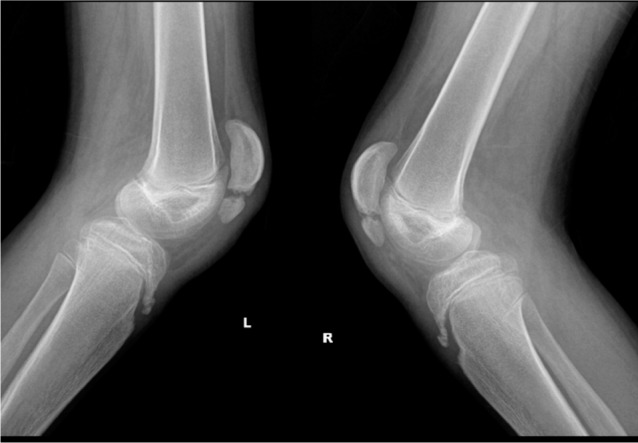
Pre-op radiograph of knee showing fixed flexion deformity with patella alta with fragmentation of distal part of patella.

He was able to crouch walk independently without walking aids. He had fixed flexion deformities (20 degrees) of both hips, and bilateral fixed flexion deformities of the knee (30 degrees) (Fig. 1, 2). The popliteal angle was 60 degrees on both sides. His iliopsoas, rectus femoris, adductors and hamstrings were contracted. He had 50 degrees quadriceps lag both sides. Torsional profile was normal both side. Patella alta with the fragmentation of the distal pole of the patella was noted on both sides. He underwent bilateral adductor, iliopsoas and rectus releases with bilateral femoral shortening with extension and patella plication. The amount of the shortening was decided intra-operatively after osteotomy of distal femur and the amount of overlap after correction of the deformity. The amount of femur shortening was 2.5 cm bilaterally. The femur shortening osteotomies were fixed with plate osteosynthesis. Neurovascular bundle was not stretched intra-operatively. He was immobilised with above knee cast in full extension for six weeks. Postoperatively, there were no vascular or neurological deficits noted. Post-operative pain was controlled with injectable analgesics for first three days, after which he did not have any severe pain.

He was found to have persistent high blood pressure ranging from 150/100 mm Hg to 180/120 mm Hg on the second postoperative day. No symptoms of increased intracranial pressure (ICP) were noted. A differential diagnosis of essential hypertension was ruled out as there was no family history of hypertension as well as the repeated pre-op blood pressures and the blood pressure readings at the start of the procedure had been found to be normal. He was investigated for secondary causes of hypertension. His renal function test and serum electrolytes including ionized calcium were normal. Thyroid function test, cortisol, urinary catecholamine, vanillyl mandelic acid, homovanillic acid, plasma renin, aldosterone level were normal. Ultrasound of the abdomen, renal artery Doppler scan, and echocardiogram were normal.

**Fig. 3:**
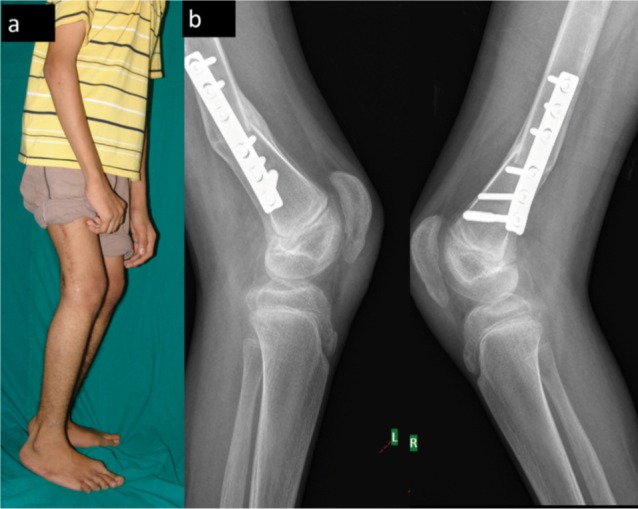
(a) Post-op standing photograph showing significant improvement of gait, and (b) Post-op radiograph of knee showing the correction fixed flexion deformity and patella alta with healing of stress fracture of the patella 1 year following surgery

He was started on oral antihypertensive medications on the third post-operative day. He was on Nifedipine 20 mg BD for 6 weeks, Clonidine 100µg 1-1/2-1/2 for 6 weeks initially. Later, he was treated with Nifedipine 20 mg BD, Metoprolol 100 mg BD and Moxonidine 0.2 mg BD. The patient was discharged two weeks after surgery and kept on periodic review, and the blood pressure showed a decreasing trend but not to normal levels. He underwent bilateral hamstring transfer (semitendinosus to the back of femur) four months following the index surgery to achieve the optimum flexion deformity correction. His blood pressure at the time of the second surgery ranged between 130/90 mm Hg and 140/100 mm Hg with antihypertensive medication. The blood pressure of the patient was higher than the value for 99th percentile for his age and sex. Following the second surgery, there was no further increase in the blood pressure. His blood pressure was controlled with a single antihypertensive medication for 15 months after surgery. At the last follow-up (15 months following index surgery), the boy was walking upright without any walking aids (Fig. 3 a,b).

## Discussion

Hypertension following flexion deformity correction and limb lengthening is well described in the literature^2-5^. Hypertension has also been reported in children treated with skeletal traction. During the last three decades, little has been written regarding post-operative hypertension following orthopaedics intervention, except for a recent report of hypertension detected post-operatively with deformity correction in a Taylor spatial frame^5^.

Shah *et al*^2^, noted the higher frequency of high blood pressure (21%) in patients with correction of severe fixed flexion deformities of the knee. The frequency of hypertension was more often noted in poliomyelitis patients than cerebral palsy. Though the frequency of hypertension was more with bilateral than unilateral corrective surgery cases, it was not statistically significant. They did not find a correlation of hypertension with the severity of the flexion deformity of the knee^2^.

Harandi *et al*^3^ reported two cases with post-operative hypertension following flexion deformity correction and postulated sympathetic stimulation as the cause for hypertension.

There are several possible mechanisms behind the unexplained hypertension. The four common ones are firstly prolonged immobilization leading to hypercalcemia; secondly increased tension in neurovascular bundle increasing the sympathetic tone; thirdly increased tension in the sciatic nerve possibly leading to indirect increased activity of sympathetic fibres and adrenal catecholamine release and fourthly sudden change in the microenvironment of the limb leading to irritability of the nerve and initiation of reflex vasoconstriction. The child in our report developed hypertension without prolonged immobilization and neurovascular bundle was not stretched. So the possibility of the first two reasons is excluded here. There may be the possibility of third and fourth reasons for development of hypertension in this child.

There is no reported case of persistent postoperative hypertension following femoral shortening osteotomy. Femoral shortening osteotomy is usually indicated for the correction of severe flexion deformity of the knees to minimize the stretch of neurovascular tissues. The aetiology of the hypertension is unclear in this case. Literatures^2-5^ note that most instances of post-operative hypertension are transient and blood pressure returns to normal after a short duration. So, we can postulate that the underlying hypertension would have been aggravated or revealed by the major surgical procedure. The uniqueness of this instance is that the hypertension persisted after continued treatment for 15 months during the post-operative period.

## Conclusion

Hypertension is a rare complication following corrective surgery for the treatment of crouch gait. Blood pressure should be checked routinely during the post-operative period to detect such a rare complication. Ruling out systemic causes is essential in such cases to prevent systemic damage. Parent’s consent had been obtained for the management of this child and there were no conflicts of interest.
